# Stabilizing Cellular Barriers: Raising the Shields Against COVID-19

**DOI:** 10.3389/fendo.2020.583006

**Published:** 2020-09-30

**Authors:** Julia Hanchard, Coral M. Capó-Vélez, Kai Deusch, Darcy Lidington, Steffen-Sebastian Bolz

**Affiliations:** ^1^Aphaia Pharma AG, Zug, Switzerland; ^2^Department of Physiology, University of Toronto, Toronto, ON, Canada; ^3^Toronto Centre for Microvascular Medicine at The Ted Rogers Centre for Heart Research Translational Biology and Engineering Program, University of Toronto, Toronto, ON, Canada

**Keywords:** glucagon like peptide 1 (GLP-1), enteroendocrine, lung, immune cells, tumor necrosis factor (TNF), tumor necrosis factor converting enzyme (TACE), endothelial barrier disruption, acute respiratory and circulatory disruption

## Abstract

The severe acute respiratory syndrome coronavirus 2 (SARS-CoV-2) and its clinical manifestation (COVID-19; coronavirus disease 2019) have caused a worldwide health crisis. Disruption of epithelial and endothelial barriers is a key clinical turning point that differentiates patients who are likely to develop severe COVID-19 outcomes: it marks a significant escalation in respiratory symptoms, loss of viral containment and a progression toward multi-organ dysfunction. These barrier mechanisms are independently compromised by known COVID-19 risk factors, including diabetes, obesity and aging: thus, a synergism between these underlying conditions and SARS-CoV-2 mechanisms may explain why these risk factors correlate with more severe outcomes. This review examines the key cellular mechanisms that SARS-CoV-2 and its underlying risk factors utilize to disrupt barrier function. As an outlook, we propose that glucagon-like peptide 1 (GLP-1) may be a therapeutic intervention that can slow COVID-19 progression and improve clinical outcome following SARS-CoV-2 infection. GLP-1 signaling activates barrier-promoting processes that directly oppose the pro-inflammatory mechanisms commandeered by SARS-CoV-2 and its underlying risk factors.

## Introduction

The severe acute respiratory syndrome coronavirus 2 (SARS-CoV-2) outbreak, first reported in December 2019 in Wuhan, China (1), rapidly evolved into a global pandemic. Comprehensive lock-down measures in most affected jurisdictions have slowed down the spread of the virus, however, in the face of the massive collateral societal and economic damage, these measures are clearly unsustainable. The core issue at hand is the lack of specific treatments for SARS-CoV-2: several antiviral strategies are undergoing clinical study, but thus far, efficacy is limited (2, 3); vaccines are in development, but these will likely not be ready in time to mitigate the current wave or prevent a second global wave. Thus, it is incumbent that we identify interventions that increase the resilience to SARS-CoV-2 infection, particularly in populations at risk of severe responses. To do so, a comprehensive understanding of the disease pathology and the risk factors that increase susceptibility for severe disease progression is required.

SARS-CoV-2 infection can be symptomless in some individuals, while on the other end of the continuum, severe cases elicit terminal multi-organ failure (4, 5). The majority of cases are mild; ~20% of cases require clinical intervention, with ~5% progressing to critically ill stages where mortality is high (5.8% global case fatality ratio, ranging from 0.1 to 16% by country) (1, 6, 7). Overall susceptibility, the likelihood of developing severe symptoms, and mortality all correlate with several known risk factors, including high BMI (>30) (8), diabetes (9–11), hypertension (8, 10) and age [corrected for comorbidities (8–10)]. In all likelihood, these risk factors compromise immune responses and/or permit systemic viral entry and replication. With regard to the latter, the SARS-CoV-2 virus is capable of compromising two critical barriers: the epithelial-endothelial barrier in lung alveoli and the vascular endothelial barrier in the systemic circulation. Indeed, alveolar-endothelial barrier failure is likely the key turning point differentiating patients who will quickly worsen into severe cases, as it marks a significant escalation in respiratory symptoms, the loss of viral containment and a progression toward multi-organ dysfunction (12–14).

This review will focus on the key cellular mechanisms that SARS-CoV-2 utilizes to disrupt epithelial and endothelial barriers. These barrier mechanisms are independently compromised by known coronavirus disease 2019 (COVID-19) risk factors; a combination effect may explain why these risk factors correlate with more severe outcomes. As an outlook, we propose a therapeutic intervention that may slow COVID-19 progression and improve clinical outcome following SARS-CoV-2 infection. In this regard, glucagon-like peptide 1 (GLP-1) signaling activates barrier-promoting processes that directly oppose the pro-inflammatory mechanisms commandeered by SARS-CoV-2 and its underlying risk factors. Thus, medications that stimulate GLP-1 signaling, e.g., exendin-4, may have unappreciated utility for COVID-19 treatment.

## Infection Mechanism

The mechanisms mediating SARS-CoV-2 infection and viral replication are already defined and will not be described in detail in this review (15–17). Briefly, host cells must express two components that are critical for SARS-CoV-2 infection: (i) angiotensin converting enzyme 2 (ACE2), the surface receptor that mediates viral attachment to the host cell, and (ii) the transmembrane serine protease TMPRSS2, which cleaves the viral spike protein, thereby priming viral fusion to the host cell's membrane (16, 18). All barrier forming cells, including lung epithelial cells (19–21), enteric epithelial cells (22, 23) and vascular endothelial cells (22) express ACE2 and TMPRSS2 in high abundance and therefore, are targeted by the SARS-CoV-2 virus. An additional element, named tumor necrosis factor converting enzyme (TACE; ADAM17), may facilitate viral entry, although the molecular mechanisms mediating the enhanced entry have not been defined (24, 25).

In the absence of an effective vaccine, intervention strategies have primarily focussed on reducing (i) SARS-CoV-2 fusion/entry, (ii) SARS-CoV-2 replication and (iii) excessive inflammation (2, 3). Obviously, preventing SARS-CoV-2 infection is more desirable than reacting to infection: thus, targeting ACE2/SARS-CoV-2 binding and TMPRSS2 activity, the crucial host proteins involved in viral entry, are highly attractive therapeutic strategies. In this regard, a clinical-grade recombinant ACE2 decoy receptor (26) and the clinically available TMPRSS2 inhibitor camostat (18) have displayed positive results *in vitro*; however, these strategies have yet to be assessed in clinical trials and are currently a long way from the patient's bedside. In fact, after months of intensive study, most medications repurposed to combat COVID-19, including the notable candidates hydroxychloroquine (27) and lopinavir-ritonavir (28), have failed to demonstrate benefit in randomized placebo-controlled clinical trials. At present, remdesivir, an adenosine nucleotide analog that hampers viral replication (29), is the only candidate (30, 31) with an active FDA emergency use authorization (EUA) at present. However, remdesivir is clearly not a *magic bullet intervention* (31) and may yet fail to demonstrate benefit in properly powered randomized placebo-controlled clinical trials.

Since targeting SARS-CoV-2 viral entry and replication has not been successful to date, “containing” the virus to the respiratory tract is of paramount importance. Since SARS-CoV-2 is predominantly transmitted through the inhalation of airborne droplets and aerosols, epithelial cells within the upper and lower respiratory tract are the first barriers to be attacked. If the virus breaches this barrier and enters the cardiovascular system, the virus will have the opportunity to infect every organ in the body via the microcirculation (32). Indeed, pronounced vascular injury in association with diffuse alveolar damage is a key feature of SARS-CoV, a relative of SARS-CoV-2 that also targets ACE2 (33).

## Possible Routes to the Systemic Circulation

In most cases, SARS-CoV-2 remains confined to the upper respiratory tract, favoring mild symptoms. Epithelial cell infection in the upper airways is associated with copious viral shedding, high person-to-person transmissibility, occasional loss of olfaction, sore throat, fever, and a characteristic dry cough. The nasal mucosa potentially provides a highly vascularized entry point to the systemic circulation if the virus can alter the properties of the restrictive tight junctions in the nasopharyngeal epithelium and underlying microvascular endothelium (34).

From the upper respiratory tract, the SARS-CoV-2 may descend down the trachea and infect cells in the lower respiratory tract and alveoli. At the bronchiolar level, SARS-CoV-2 can infect epithelial goblet cells (35), resulting in airway inflammation and mucous secretion. The inflammatory response subsequently impairs mucociliary clearance, which hampers the clearance of the viral particles, and elicits complications such as bronchiectasis and bronchial wall thickening (36). At the alveolar level, infection and subsequent disruption of the “blood-air barrier,” which comprises alveolar epithelial cells and pulmonary microvascular endothelial cells, is a central event in disease progression: in essence, it is a transition point from relatively moderate symptoms to the severe respiratory symptoms and lung injury observed in severe COVID-19 cases. The compromised barrier becomes leaky, permitting alveolar fluid accumulation (edema), and the development of pneumonia and inflammatory cell infiltration. The resulting hypoxia and damage unleashes a “cytokine storm” in a subset of patients that perpetuates a vicious cycle of progressive lung injury, as the inflammatory response further damages pulmonary cells and compromises endothelial function and barrier integrity (37). In addition to driving severe lung injury, the breach of the blood-air barrier permits viral entry into the systemic circulation, where the virus can then cause widespread multi-organ damage (12, 14).

## Tace Is a Key Driver of SARS-COV-2 Severity

Not all coronavirus infections elicit severe respiratory system injury and multiorgan damage: for example, HNL63-CoV, a coronavirus that also binds to ACE2 (38), generally causes relatively mild common cold symptoms (39). Although HNL63-CoV and SARS-CoV-2 bind to the same surface receptor, a key difference between the two viruses resides in the activation of TACE: SARS-CoV strongly activates TACE sheddase activity (24, 25), while HNL63-CoV does not (24). This suggests that TACE activation is a key underlying aspect of the SARS-CoV-2 disease severity.

**Figure 1:**
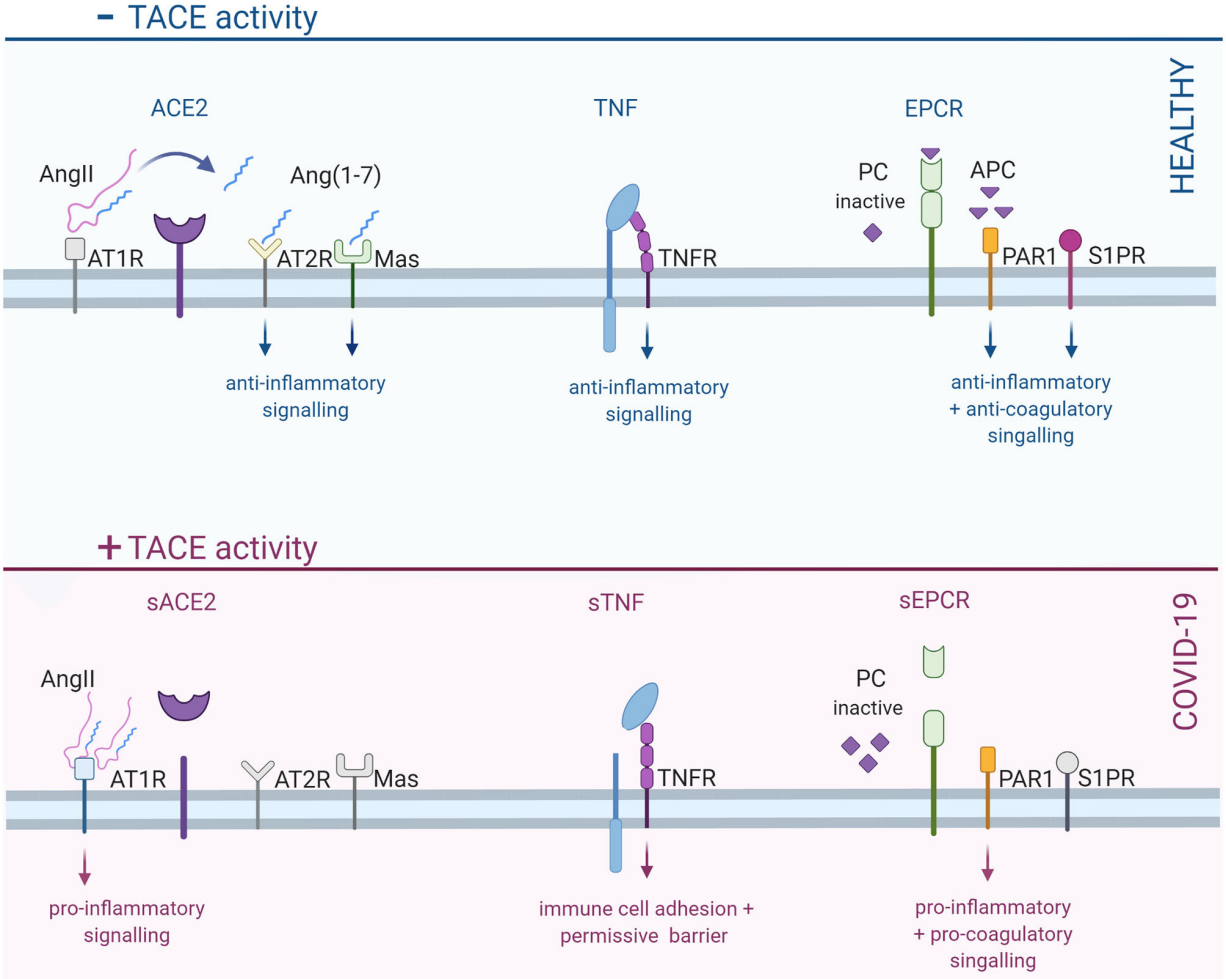
Increased TACE activity in COVID-19 shifts barrier cells toward a pro-inflammatory, pro-coagulatory and permissive state. In healthy cells, TACE is predominantly inactive, thereby permitting anti-inflammatory signals via TACE substrates, including ACE2, TNF, and EPCR. ACE2, a key player in the RAAS system, converts angiotensin II (AngII) into angiotensin-1-7 [Ang (1–7)]: the subsequent activation of AT2R and Mas receptors stimulates anti-inflammatory signals. When ACE2 is cleaved, angiotensin signaling shifts from AT2R/Mas receptors to the pro-inflammatory AT1R. TNF receptors stimulate differential signals, depending on whether the receptor is activated by the membrane-bound or soluble form of TNF: membrane bound TNF elicits important anti-inflammatory signals; in contrast, soluble TNF mediates pro-inflammatory signals. Finally, EPCR plays a critical role in regulating coagulation by converting inactive protein C (PC) into activated protein C (APC). Activated protein C subsequently inactivates pro-coagulatory factors; it also cleaves PAR1 and transactivates S1PR, resulting anti-inflammatory and barrier-stabilizing signals. TACE-dependent EPCR cleavage prevents the activation of PC, leading to reduced capacity to inhibit coagulation; RhoA-dependent signaling via uncleaved PAR1 is barrier destabilizing. TACE, tumor necrosis factor alpha converting enzyme; ACE2, angiotensin converting enzyme 2; TNF, tumor necrosis factor; EPCR, endothelial protein C receptor; RAAS, rennin-angiotensin-aldosterone system; AT2R, angiotensin receptor 2; AT1R, angiotensin receptor 1; PAR1, protease activated receptor 1; S1PR, sphingosine-1-phosphate receptor; TNFR, TNF receptor. Created with BioRender.com.

TACE has more than 80 known substrates, including growth factors, cytokines, cell surface receptors and adhesion molecules (40) and hence, plays complex roles in many regulatory processes (40): thus, it is not surprising that perturbing normal TACE function yields a broad spectrum of deleterious effects. In the context of the COVID-19 pathology, three particular TACE substrates stand out: ACE2 (41), tumor necrosis factor (TNF) (4, 42), and the endothelial protein C receptor (EPCR) (43). All three of these proteins play important anti-inflammatory and barrier-stabilization roles: TACE-dependent shedding of these cell surface proteins, therefore, shifts a delicate balance in favor of inflammation and reduced barrier integrity (Figure 1).

The physiological functions of ACE2 and the implications of ACE2 shedding in COVID-19 have been extensively reviewed by Gheblawi et al. (41). ACE2 is a central element in the Renin-Angiotensin-Aldosterone System (RAAS) and therefore, has wide ranging effects that intersect with virtually every endocrine and inflammatory mechanism (44). At the molecular level, ACE2 converts angiotensin II into angiotensin (1-7): angiotensin II activates the pro-inflammatory angiotensin II receptor subtype 1 (AT1R) (45), while angiotensin (1-7) preferentially activates the anti-inflammatory angiotensin II receptor subtype 2 (AT2R) (46) and Mas receptors (46–48). Thus, TACE-dependent ACE2 shedding (41, 49) in COVID-19 shifts RAAS signaling in favor of the pro-inflammatory AT1 receptors (50), which favors immune cell adhesion (51), cellular damage (51), and increased vascular permeability (via the modulation of VE-cadherin function) (52, 53).

Although TNF is best known as a pro-inflammatory cytokine, TNF serves many developmental, homeostatic and reparative functions (54–56). In pathological settings, TNF is a critical initiating factor in the immune response; importantly, if the initial pathogenic insult and/or tissue damage is severe, the immune response can spiral out-of-control into the highly damaging “cytokine storm” (54–58). At the level of the endothelium, TNF stimulates two key homeostatic changes: (i) the expression of immune cell adhesion molecules (e.g., VCAM-1 and ICAM1) (59, 60), and (ii) an increase in barrier permeability, via cytoskeletal rearrangement (61, 62) and the regulation of cell-to-cell adhesion junctions (63, 64). These changes allow immune cells to bind to the endothelium at the site of injury/infection and transmigrate into the tissue through the paracellular junctions (54, 58). Among other notable acute effects, TNF also simulates endothelial reactive oxygen species generation and impairs nitric oxide production (54), which can have significant effects on tissue damage and vascular control mechanisms (65, 66). In COVID-19, TACE-dependent TNF shedding favors the rapid breakdown of the endothelial-alveolar barrier, resulting in lung edema, immune cell infiltration, and ultimately, lung tissue damage. This barrier breakdown also opens the gateway to the systemic circulation: once distal endothelial cells are infected; the same inflammatory mechanism provides a means for the virus to escape the systemic circulation and into organ parenchymal cells.

The endothelial protein C receptor (EPCR) (67, 68) is best known for its anti-coagulatory functions (69); however, EPCR signaling also plays an important role in moderating inflammation (70–72), maintaining endothelial barrier function (73, 74) and conferring cytoprotection (75–77). EPCR binds the zymogen protein C and cleaves it into an active protease: this activated form of protein C remains bound to the EPCR and subsequently (i) cleaves protease-activated receptor 1 (PAR1) and (ii) transactivates the sphingosine-1-phosphate receptor 1 subtype (S1PR_1_) (67, 68). The activation of these receptors inhibits nuclear factor-κB (NF-κB) translocation/signaling, thereby reducing proinflammatory gene expression, the release of cytokines, and the expression of adhesion molecules (67, 68). In addition, activated protein C shifts PAR1 signals from RhoA-dependent, barrier permeabilizing actions to Rac1-dependent, barrier stabilizing actions (67, 68). In the context of COVID-19, TACE-dependent EPCR shedding removes an important molecular brake that dampens immune cell infiltration, edema and tissue damage; by eliminating a barrier stabilization mechanism, the loss of EPCR signaling also undermines viral containment. Finally, deficient EPCR signaling, a key brake element in the coagulation pathway, likely also contributes to the high incidence of thrombotic complications observed in serious COVID-19 cases, including widespread microthrombi, pulmonary embolism, stroke and disseminated intravascular coagulation (12, 78, 79).

In summary, the activation of TACE may significantly augment the inflammatory response following SARS-CoV-2 infection. As a component of the inflammation, endothelial permeability may become severely compromised, permitting the SARS-CoV-2 virus with access to the systemic circulation. Given the remarkably large surface area of the microvascular endothelium (3,000-4,000 m^2^) and its presence in every tissue, the failure to maintain this barrier eliminates the last line of defense against multi-organ damage and failure.

## COVID-19 Risk Factors That Compromise Barrier Function

Several cardiovascular risk factors, including hypertension, obesity, type 2 diabetes mellitus (T2D) and cardiovascular disease, are common underlying conditions in COVID-19 patients (80, 81). Although these risk factors tend to associate with more severe cases, the individual contribution of each risk factor to COVID-19 disease severity/outcome is difficult to define, due to the small sample size of most studies, combined with the fact that these risk factors largely overlap [e.g., 52% of patients with T2D are obese; and 60% of obese patients have metabolic syndrome including hypertension as a leading symptom (82–85)]. To add a further challenge, age is a significant COVID-19 risk factor (86) that has generally not been taken into account in most risk factor studies (81). According to Tadic et al. (81), most risk assessment studies are too small, inconsistent, and fail to account for several confounding factors, most notably age and obesity: consequently, they are ill-equipped to discern important interactions between comorbidities and outcomes. Thus, the data are more epidemiological than analytical and should be viewed cautiously (81).

Although the precise relationships between COVID-19 risk factors and disease severity require clarification, there are obvious mechanistic commonalities across these risk factors that permits speculation as to why certain common underlying conditions appear to cluster with more severe outcomes. In this sense, we can predict that the outcomes should be more severe, based on deleterious mechanisms that are already activated prior to infection. As a prime example, metabolic conditions such as obesity and T2D down-regulate tissue inhibitor of metalloproteinase 3 (TIMP3), an important endogenous TACE inhibitor that critically regulates TACE and the release of TNF in metabolic tissues (87, 88). The resulting increase in TACE activity induces a proinflammatory state (e.g., cytokine production, immune cell adhesion molecule expression, and hyperpermeability) and pronounced endothelial dysfunction (e.g., reactive oxygen species generation and impaired autacoid release) (89–92) observed in obese and T2D patients. Aging is a double-hit: in addition to inducing pro-inflammatory endothelial dysfunction (93, 94), it also diminishes infection defense (“immune senescence”) (95, 96).

A common thread across these risk factors is chronic low-level inflammation and endothelial dysfunction: these risk factors facilitate infection, prime an exaggerated immune response and/or compromise viral containment. Thus, it is entirely reasonable to expect that these underlying conditions would synergize with SARS-CoV-2 infection mechanisms to more profoundly erode barrier function and drive more severe disease progression. These underlying conditions also profoundly impact the patient's immunological status, which is an obvious determinant of COVID-19 severity (97). Indeed, deep immune profiling reveals that hospitalized patients fall across a spectrum of immune response patterns (98), with underlying conditions contributing to the diversity in host responses.

## GLP-1 Agonists: a Potential Intervention for Mitigating COVID-19

Glucagon-like-peptide 1 (GLP-1) is an enteroendocrine hormone, originally characterized in the orchestration of insulin release in response to ingested nutritional stimuli (99, 100). However, GLP-1 signaling plays a vital role in energy metabolism and cell viability in several tissues: thus, targeting GLP-1 receptors can potentially elicit systems-level effects (101). In this regard, GLP-1 has emerged as an important homeostatic element within the cardiovascular system, where it possesses significant endothelial-protective functions (102, 103). It is also interesting to note that all of the barrier-forming cells in the lung and vascular system express GLP-1 receptors (104–108).

In the lung, GLP-1 tightens barriers via the upregulation of tight junction proteins in barrier-forming cells (108, 109); in alveolar type 2 pneumocytes, GLP-1 stimulates the production of surfactant that, by reducing surface tension, helps to minimize fluid accumulation within alveolar spaces (110). In endothelial cells, GLP-1 inhibits TACE expression and activity (111): it therefore directly opposes key mechanisms that SARS-CoV-2 commandeers to augment inflammation and compromise barrier function. Accordingly, GLP-1 signaling attenuates TACE-dependent EPCR shedding (111); GLP-1 signaling also increases deficient ACE2 levels in pathological settings (112), presumably via reduced ACE2 shedding. Consistent with this anti-inflammatory role, GLP-1 receptor agonists possess several desirable actions, including (i) antagonizing inflammatory NF-κB signaling (103), (ii) reducing immune cell adhesion molecule expression on endothelial cell surfaces (e.g., ICAM-1 and VCAM-1) (103, 113), (iii) reducing immune cells cytokine production (113), and (iv) attenuating endothelial cell oxidative stress (114).

**Figure 2:**
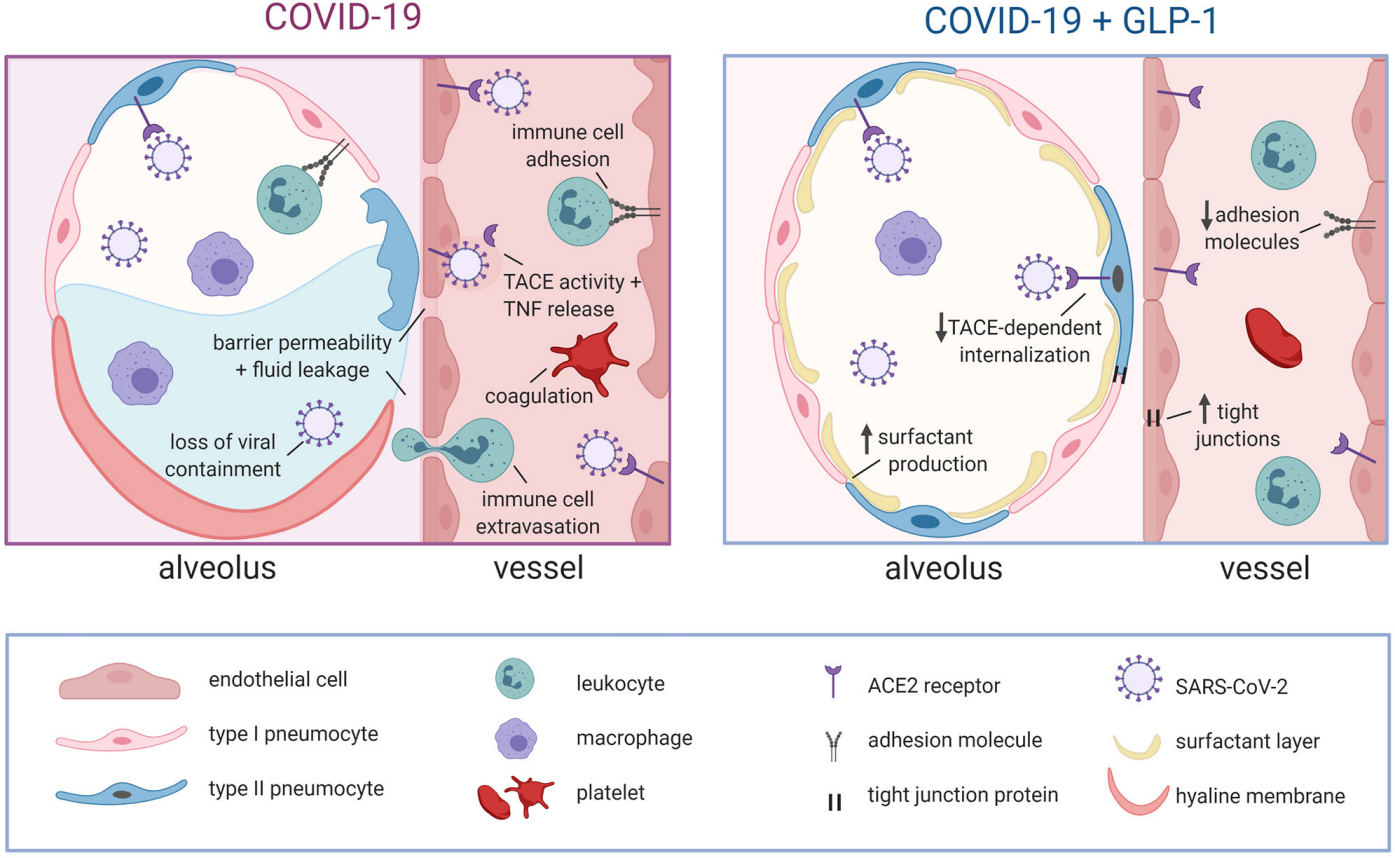
Proposed mechanisms for GLP-1-mediated stabilization of alveolar epithelial and endothelial barriers in COVID-19. In COVID-19, alveolar epithelial cells are severely damaged by direct viral attack and the immune response, leading to hyaline membrane formation, increased barrier permeability, alveolar fluid accumulation and a loss of viral containment. Viral attack on the underlying endothelial cells leads to TACE activation that: augments pro-inflammatory signaling, increases endothelial barrier permeability, promotes immune cell adhesion/extravasation and compromises EPCR-dependent regulation of coagulation. Notably, the loss of endothelial barrier integrity further exacerbates alveolar fluid accumulation. GLP-1 receptor signaling directly opposes these pathological mechanisms by: attenuating TACE expression/activity, initiating counteracting anti-inflammatory signals, promoting barrier function via increased tight junction expression and stimulating surfactant secretion (reduces alveolar fluid accumulation by decreasing surface tension). The GLP-1 mediated attenuation of TACE activity/expression is also expected to hinder viral entry into cells. GLP-1, glucagon like peptide 1; ACE2, angiotensin converting enzyme 2; TACE, tumor necrosis factor alpha converting enzyme; TNF, tumor necrosis factor; EPCR, endothelial protein C receptor. Created with BioRender.com.

With regard to the risk factors associated with severe COVID-19 cases, it is remarkable to note that GLP-1 receptor agonists (e.g., exendin-4, liraglutide, semaglutide) are either FDA-approved (diabetes) (115) or proposed (obesity, age-related decline) (116, 117) as an intervention for the underlying conditions. GLP-1 receptor agonists were specifically developed to harness GLP-1's potent hypoglycemic effect in the treatment of diabetes (115): the improved glycemic control results in weight loss in T2D patients and consequently, clinical trials are currently assessing their utility as an anti-obesity drug for patients without T2D (116). In addition to its functions as a metabolic hormone, GLP-1 possesses significant anti-inflammatory effects that are independent of glucose homeostasis (118). Consequently, GLP-1 receptor agonists are now being considered for age-related pathologies, including Alzheimer's disease, Parkinson's disease, and cognitive decline, all of which possess a strong inflammatory component (117, 119). Since GLP-1 signaling exerts clear beneficial effects in obesity, T2D and aging, it must potently ameliorate a common pathological thread across these conditions: chronic low-level inflammation and endothelial dysfunction. Since (i) both the risk factors for severe COVID-19 (e.g., obesity, diabetes, age) and the SARS-CoV-2 virus harness similar pro-inflammatory mechanisms and (ii) GLP-1 signaling is anti-inflammatory and has demonstrable benefits in patients with underlying conditions (118), it stands to reason that GLP-1 signaling should directly oppose the inflammatory mechanisms activated in COVID-19. In this context, GLP-1 agonists would be a useful mechanism-based treatment strategy (Figure 2).

## GLP-1 Secretagogues: a Novel Opportunity for Prophylaxis

Given that GLP-1 signaling confers many positive benefits, especially in individuals with COVID-19 risk factors (118, 120, 121), it is intriguing to hypothesize that the benefits of GLP-1 can be harnessed prophylactically. In essence, the objective would be to activate the endogenous barrier-promoting and anti-inflammatory actions of GLP-1 signaling prior to SARS-CoV-2 infection, with the prospect that this would increase resilience in the event of infection. Based on the rapid and dramatic metabolic effects observed in Roux-en-Y Gastric Bypass (RYGB) patients (122, 123) there is no doubt that the intestinal tract has the enteroendocrine capacity to drive significant beneficial effects on the systemic level. Further, extreme measures are not necessary to elicit systemic effects: even normal, post-prandial GLP-1 secretion is sufficient to stimulate nitric oxide production in the forearm, thereby increasing blood flow and oxygen uptake (124, 125). The fact that “normal” enteroendocrine GLP-1 signaling activates the endothelium is important, because it suggests that the full repertoire of positive GLP-1 effects may be in play. Thus, if endogenous GLP-1 signaling could be stably or perpetually activated, it may be possible to increase endothelial and epithelial “resilience” to SARS-CoV-2 infection.

Eliciting prophylactic GLP-1 release from the intestine is likely mechanistically simple, extremely safe, and in all probability, very low cost. Virtually any carbohydrate, lipid or protein macronutrient could be used as a candidate secretagogue, as these nutrients clearly mobilize GLP-1 secretion mechanisms (126–128). Assuming that secretagogues stimulate sufficient GLP-1 release to positively influence inflammatory and barrier-promoting mechanisms at the systemic level, there would be little argument that these stimuli would: (i) be safe to ingest, as they are basic food components, (ii) not cause the adverse effects associated with the supra-physiological levels of GLP-1 signaling elicited by GLP-1 agonists, such as headache, vomiting or diarrhea (129), (iii) be cost effective to manufacture and purchase and (iv) have immediate worldwide availability. This strategy is obviously speculative and not currently available for use against COVID-19; it is nevertheless worth pursuing, as it could have broad implications for patients with obesity, T2D, cardiovascular disease and other pathologies that target the endothelium, inflammatory mechanisms or barrier function.

## Summary and Outlook

In summary, the breakdown of cellular barriers is a key driver of severe SARS-CoV-2 disease progression. The underlying pro-inflammatory mechanisms that disrupt barrier function in COVID-19 are well-characterized and substantially overlap with the disease mechanisms operating in diabetes, obesity and aging, all putative COVID-19 risk factors. Since anti-inflammatory interventions that strongly supress immune function (e.g., anti-TNF therapeutics) are not recommended for treating COVID-19, we need to deploy other options that interfere with these barrier-disrupting mechanisms. GLP-1 is an intriguing candidate, because it possesses both anti-inflammatory and barrier-promoting properties. Indeed, GLP-1 signaling is currently proposed as an intervention for the very risk factors that also drive aggravated COVID-19 severity. It is, therefore, tempting to speculate that GLP-1 signaling could be harnessed to fight COVID-19 on two levels: secretagogues could prophylactically increase the global population's resilience to the infection, and in acute COVID-19, GLP-1 receptor agonists may be useful in supporting acute therapeutic interventions.

## Author Contributions

JH, CC-V, and S-SB conceived this review. JH and CC-V contributed equally to the literature research and figure preparation. JH, CC-V, KD, DL, and S-SB all significantly contributed to writing and revising the manuscript.

## Conflict of Interest

S-SB and KD are executive board members of Aphaia Pharma AG, JH and CC-V are employees of Aphaia Pharma AG, and DL is a paid consultant for Aphaia Pharma AG.
